# Comparative Analysis of YOLO Series Algorithms for UAV-Based Highway Distress Inspection: Performance and Application Insights

**DOI:** 10.3390/s25051475

**Published:** 2025-02-27

**Authors:** Ziyi Yang, Xin Lan, Hui Wang

**Affiliations:** 1 College of Artificial Intelligence, Southwest University, Chongqing 400715, China; zy_yanghhh@163.com (Z.Y.); lanxinwdls@163.com (X.L.); 2 Key Laboratory of New Technology for Construction of Cities in Mountain Area, Ministry of Education, School of Civil Engineering, Chongqing University, Chongqing 400045, China

**Keywords:** YOLO series algorithms, UAV-based inspection, highway distress

## Abstract

Established unmanned aerial vehicle (UAV) highway distress detection (HDD) faces the dual challenges of accuracy and efficiency, this paper conducted a comparative study on the application of the YOLO (You Only Look Once) series of algorithms in UAV-based HDD to provide a reference for the selection of models. YOLOv5-l and v9-c achieved the highest detection accuracy, with YOLOv5-l performing well in mean and classification detection precision and recall, while YOLOv9-c showed poor performance in these aspects. In terms of detection efficiency, YOLOv10-n, v7-t, and v11-n achieved the highest levels, while YOLOv5-n, v8-n, and v10-n had the smallest model sizes. Notably, YOLOv11-n was the best-performing model in terms of combined detection efficiency, model size, and computational complexity, making it a promising candidate for embedded real-time HDD. YOLOv5-s and v11-s were found to balance detection accuracy and model lightweightness, although their efficiency was only average. When comparing t/n and l/c versions, the changes in the backbone network of YOLOv9 had the greatest impact on detection accuracy, followed by the network depth_multiple and width_multiple of YOLOv5. The relative compression degrees of YOLOv5-n and YOLOv8-n were the highest, and v9-t achieved the greatest efficiency improvement in UAV HDD, followed by YOLOv10-n and v11-n.

## 1. Introduction

**Figure 1:**
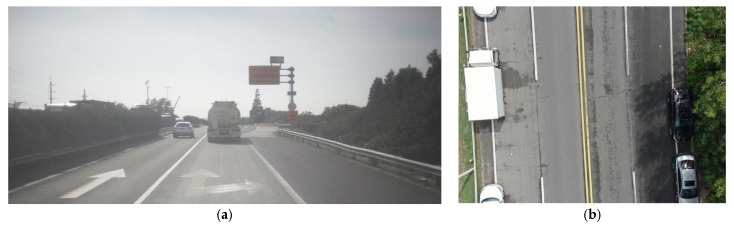
Comparison of image data shooting range. (**a**) Vehicle-mounted vision, self-collected; (**b**) UAV photography (15 m width), produced based on dataset [17].

As a sensor-bearing platform, an unmanned aerial vehicle (UAV) is equipped with soulful shapes and free shooting angles that can be better adapted to the needs of different application scenarios [1]. The conjunctive application of UAVs with deep learning algorithms has been a subject of considerable interest in infrastructure management. In the field of visual sensors for infrastructure anomaly detection, the YOLO (You Only Look Once) family has emerged as the most promising detection algorithm [2]. UAVs have been gradually applied to pavement inspection over the years [2,3,4,5,6,7,8,9] but have not been used on a large scale due to flight range and batteries. In-vehicle cameras are still the mainstay of pavement distress and facility detection [10,11,12,13,14,15]. However, vehicle-mounted cameras often have problems such as a small field of view and the inability to cover the cross-section, as shown in Figure 1. These problems may result in structural damage not being detected. Spatial intelligence technology based on UAV inspections brings practical technical support for the one-time detection of safety problems including through-type cracks, large-area subsidence, and damage to retaining walls or slopes. However, UAV inspection still has problems such as restrictions on navigation routes [16], short working hours, untimely data transmission processing, and computational analysis.

The YOLO family of models has attracted considerable attention due to its real-time and efficient performance. The miniatures or small models can be released to end devices and applied to real-time detection. However, as the learning depth of the model decreases, both the detection accuracy and the complexity of the knowledge learned decrease more significantly. Hou et al. [3] demonstrated that YOLOv3 exhibited superior detection efficiency and precision compared to Faster R-CNN. Ma et al. [4] enhanced the YOLOv3 algorithm with an accelerated module, resulting in the development of a compact model that is 5–6 times faster than the original. The model was integrated with a smart inspection device and an automated UAV, allowing for the real-time detection and counting of cracks. Situ et al. [18] developed a 2.84 MB model integrating the YOLOv5 model with transfer learning and channel pruning, significant advancements have been made by researchers towards real-time target detection within the domain of fire detection. Huang et al. [19] proposed a YOLO-LNet model that surpasses the performance of YOLOv5s for forest fire detection. Computer-based tests demonstrated that the model exhibited acceptable detection accuracy, where the speed, computational quantity, and parameter size were 110 FPS (frames per second), 0.7 G FLOPs (floating point operations), and 0.26 M, respectively. The above model size and efficiency metrics for fire detection studies represent the requirements for real-time and embedded models for UAV aerial photography detection. Scenarios that require higher detection accuracy often cannot be balanced with efficiency. Guan et al. [20] proposed an enhanced YOLOv7-DRM model for UAV-based bridge defect detection and conducted a fine-tuning and KD to reduce the model volume. However, the model volume was only reduced to 32.2 M, which is even larger than that of YOLOv5s.

For road distress detection, we proposed the YOLOv7-RDD model with a 6.04 M parameter size [13]; however, the model exhibited a high overhead in embedding scenarios and its practical application was found to be challenging. The YOLOv7-RDD model was subsequently tested and compared using a publicly available UAV dataset containing four types of distress, achieving a higher level of performance than Faster R-CNN, YOLOv5s, and YOLOX-s [9]. The efficacy of PKD models is contingent upon the performance of the trained teacher model [21]. Zeng and Zhong [22] proposed YOLOv8-PD (pavement distress), achieving marginal improvements in accuracy, and significantly reduced parameter size and FLOPs. Zhang et al. proposed SMG-YOLOv8 [23], a model capable of precise pavement detection with a decreased model size, albeit at the cost of higher FLOPs. In a similar vein, Li et al. constructed an RDD-YOLOv8 [24] model based on YOLOv8, which exhibited enhanced detection accuracy and a marginal decline in model size and complexity. Zhu et al. [6] conducted a comprehensive and detailed analysis of the various parameters associated with UAV detection, including factors such as flight environment, flight altitude, image resolution, and shutter speed. With their dataset-UAPD (UAV asphalt pavement distress dataset), a mean average precision with an intersection over union above 0.5 (mAP@0.5) of 56.6% was achieved. However, the authors did not analyze the size or efficiency of the model. Moreover, they expanded the scope of UAV detection and constructed a dataset of multiple roadway anomalies (UAVROAD), achieving a mAP@0.5 of 61.9% and FLOPs of 7.9 G with an improved YOLOv8n [7].

**Figure 2:**
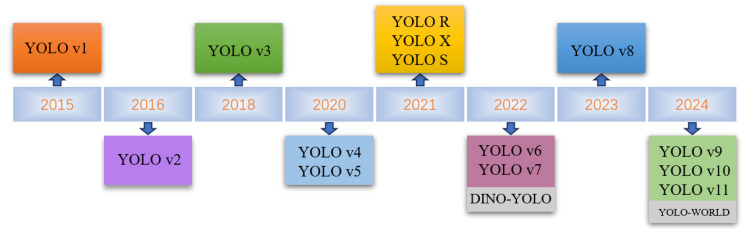
Evolution of the YOLO series of object detection algorithms.

Since YOLOv1 [25], the YOLO series of object detection algorithms has been updated from YOLOv1 to YOLOv11 [25,26,27,28,29,30,31,32,33,34,35], as shown in Figure 2. The model performance still faces significant challenges in the task of less-sample target detection. The application of the series of YOLO models to UAV highway inspection scenarios and the role played by the improvement of each part of the modules need to be systematically compared and discussed. Accordingly, this paper proposed the implementation of comparative tests of varying scales of YOLO series models based on the dataset UAV-PDD2023 [17]. The improvements of each version of the model and its applicable scenarios were analyzed by systematically comparing the structure, detection mechanisms, and performances in a less-sample detection of the YOLO series.

## 2. Methods and Dataset

### 2.1. Comparison of YOLO Family

In terms of data enhancement and training strategies, the YOLO series models continue to innovate to improve detection capability. YOLOv3 [27] uses a multiscale prediction mechanism to improve the detection accuracy through different levels of feature fusion, but the model’s adaptability is weaker on low-shot data categories, which leads to a high missing detection rate. Both YOLOv5 [29] and YOLOv7 [30] employ a variety of advanced techniques and methods in the training process, such as Mosaic data enhancement, adaptive anchor frames, gradient cropping, and multi-scale training, to improve the training efficiency and detection accuracy of the models. In addition, YOLOv7 also adopts the hybrid precision training technique, which further reduces the training time and memory usage. Compared to YOLOv7, YOLOv8 [31] offers a wide range of pre-trained models using, for example, more advanced data enhancement techniques, Mosaic 4.0 and MixUp 2.0. YOLOv9 [32] and YOLOv10 [33] both combine adaptive training strategies and online data enhancement techniques, which enable the model to better adapt to changing data distributions during training. YOLOv11 [34] combines dynamic data enhancement, distributional balanced enhancement strategies, and semi-supervised learning strategy techniques to further improve the model’s detection ability in complex scenes. YOLO-World [35] integrates a variety of state-of-the-art data enhancement methods, including Mosaic, MixUp, CutMix, random cropping, color dithering, and random rotation strategies. These methods enhance the generalization ability of the model in different dimensions by diversely modifying the training samples.

For the backbone network, there are significant differences between different versions of the YOLO backbone network in the feature extraction and processing of complex scenes. YOLOv3 [27] uses Darknet−53 as the backbone network to capture multi-scale target features through deep convolutional layers, which is suitable for the detection task in simple scenes, but the performance is not good enough when dealing with low-shot data, and is prone to missing detection. YOLOv5 [29] adopts CSPDarknet, which is based on Darknet53 and improved by introducing the CSP (Cross Stage Partial connections) structure. The CSP structure enhances the feature representation by splitting the input feature map into two parts, one part is directly passed to the next layer, the other part undergoes convolution operation, and then the two parts of the feature map are added or spliced element by element. The YOLOv7’s [30] backbone network employs E-ELAN, an enhanced version of ELAN (Efficient Layer Aggregation Network) architecture. The primary function of E-ELAN is to enhance the efficacy and efficiency of the model by optimizing the aggregation of features and computational pathways. Moreover, YOLOv7 optimizes CSPNet based on YOLOv4 by reducing redundant computation and enhancing feature representation, which makes the model more adaptable when dealing with low-shot data and performs better in embedded and resource-constrained environments. YOLOv8 [31] employs the C2f (CSPDarknet53 to 2-Stage Feature Pyramid Network) module to enable more efficient residual learning and uses the Spatial Pyramid Pooling Fast (SPPF) module to facilitate the integration of key information from targets of various sizes by combining feature maps through pooling operations at different scales. YOLOv9 [32] employs the newly proposed Programmable Gradient Information PGI and the more efficient and versatile network Generalized ELAN, both of which together form a completely new network architecture. YOLOv10 [33] further optimizes the network architecture based on YOLOv9 by combining a lightweight convolutional neural network and a multi-scale feature fusion strategy, which enhances the accuracy and depth of the feature extraction, and results in a more robust and efficient detection of complex backgrounds and small-sample categories. YOLOv11 [34] adopts a feature extraction module based on Transformer, which can better capture globally dependent features than traditional convolutional neural networks and is especially suitable for handling complex scenes and long-distance-dependent features, which makes the model perform better in detecting low-shot data categories. YOLO-World [35] combines the visual Transformer and convolutional neural networks; through the hybrid feature extraction module, it achieves a more efficient feature expression capability when dealing with multi-targets and complex scenes, and especially performs well in low-shot data categories and small-target detection.

The improvement of the loss function is also an important factor in enhancing the detection performance of the YOLO series. YOLOv3 [27] uses the GIoU loss function to enhance the regression accuracy of the bounding box but suffers from the problem of inaccurate localization. YOLOv7 [30] introduces the CIoU loss function, which improves the localization accuracy by further taking into account the overlap of the bounding box, the distance from the centroid, and the scale consistency. YOLOv8 [31] uses the Wise-MPDIoU (Modified Panoptic Distance IoU) loss weighting mechanism, which dynamically adjusts the loss weights according to the importance of different targets and significantly improves the detection accuracy of the less-sample category. YOLOv9 [32] adopts an improved CIoU loss function and introduces a weighting strategy for the less-sample category to ensure that in the case of the bounding box, the model achieves stable detection results in the presence of imbalanced positioning accuracy and categories. Compared with the previous version, YOLOv9 has significantly improved the detection performance for rare targets. YOLOv10 [33] combines the GIoU and Focal loss functions to better balance the accuracy and stability in complex scenarios, especially when dealing with small targets and sparse categories. YOLOv11 [34] introduces an adaptive loss function mechanism, which dynamically adjusts the loss function according to the target features, enhancing the model’s performance in complex environments. YOLO-World [35] employs a hybrid loss function, including both IoU and classification loss, to ensure the accurate detection of complex scenarios and few-sample categories, while maintaining the robustness of the model.

As aspects of feature fusion and detection head processing, YOLOv3 generates multi-scale output by fusing features across layers. Instead of directly using a standard Feature Pyramid Network (FPN), it generates multiscale output by combining feature maps from different layers. The detection head consists of multiple convolutional layers and uses three convolutional layers of different scales to detect large, medium, and small objects. The neck structure of YOLOv5 mainly uses the feature pyramid network (FPN) for feature fusion. It fuses feature maps of different scales through up-sampling and splicing operations to enhance the detection of objects of different sizes. The main differences between the neck of YOLOv7 and YOLOv5 are that the SPPF is replaced with SPPCSP, the CSP module is replaced with the ELAN-W module, the downsampling becomes the MP−2 layer, and the Conv2d becomes the RepBlock + CBM. Both the YOLOv5 and YOLOv7 detection headers support a variety of improvement mechanisms. YOLOv8 fuses multi-scale feature map outputs from the backbone, utilising Feature Pyramid Network (FPN) and Path Aggregation Network (PAN) concepts to construct a feature pyramid. The head network of YOLOv8 employs decoupled detection heads, using two parallel convolution branches to compute the regression and classification losses separately. YOLOv9 is more flexible in feature fusion, and YOLOv10 and YOLOv11 introduce an enhanced feature fusion module and multi-attention mechanism, respectively, which make the model perform more stably and efficiently in multi-target detection with fewer sample categories and complex environments. YOLO-World, on the other hand, enables the model to better understand complex targets in complex environments through the global feature fusion strategy, especially for multi-target detection scenarios. The model and framework chosen for the comparison tests are shown in Table 1.

As illustrated in Table 1, certain frameworks (YOLOv5, YOLOv8, YOLOv11, and YOLO-world) employ model compression predominantly through smaller depth and width multipliers, while alternative versions of the compressed model affect enhancements or modifications at the level of the backbone or neck network modules for other frameworks (YOLOv7, YOLOv9, and YOLOv10).

### 2.2. Dataset

**Figure 3:**
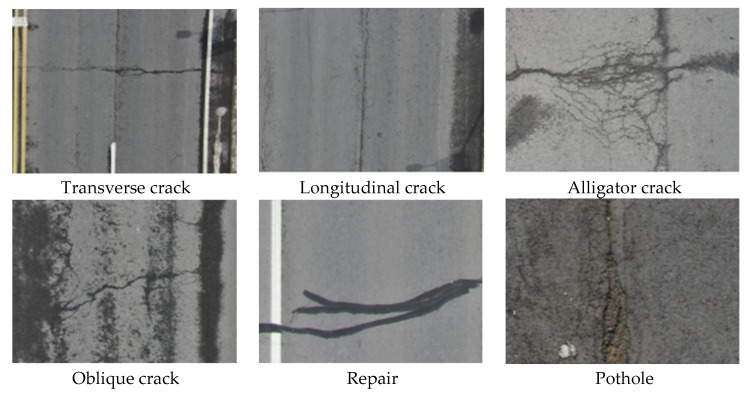
Samples of highway distress in UAV-PDD 2023 dataset [17].

The UAV-PDD2023 [17] dataset consists of road pavement images taken during UAV inspections. The dataset consists of 2425 images in JPG format with corresponding annotated files in VOC format. The pavement distress images were collected during the day, and a wide variety of weather and lighting conditions were taken into account when capturing the images. The images are 2592 × 1944 pixels in size, derived from large-format slices of images taken with a 4K camera, with four images taken at each location (top–left, top–right, bottom–left, and bottom–right). Figure 3 illustrates the samples of highway distresses in the UAV-PDD 2023 dataset. The dataset used in this paper includes six types of road distress: 603 alligator cracks (AC), 2992 longitudinal cracks (LC), 282 repairs (RP), 199 potholes (PH), 1686 oblique cracks (OC), and 5396 transverse cracks (TC), totaling 11,150 labels. The dataset is divided into training and validation sets in a ratio of 7:3. The test set includes 201 ACs, 852 LCs, 86 RPs, 60 PHs, 495 OCs, and 1627 TCs, totaling 3321 labels.

## 3. Experiment Setting and Evaluation Metrics

The experimental environment is based on Python 3.10.8, a Pytorch 2.0.1 + cu117 framework, CUDA11.7, and the hardware devices are AMD Ryzen 7 5800 H with a Radeon Graphics Processor, and NVIDIA GeForce RTX 4090.

In this paper, the mean Average Precision (mAP) and F1 score were used to evaluate the model’s accuracy. Frame rate per second (FPS) was used to evaluate the detection efficiency. The number of parameters (Params) and the number of floating-point operations per second (FLOPs) were used to evaluate the model size and complexity. Moreover, the training time (TT) was used to assist in the assessment of the lightweight degree of the model. The related evaluation metrics are calculated according to Equations (5)–(9).

(1) $$\mathrm{Precision}=\frac{TP}{TP+FP}$$

(2) $$\mathrm{Recall}=\frac{\mathrm{TP}}{\mathrm{TP}+\mathrm{FN}}$$

(3) $$F1=2\times \frac{\mathrm{Precision}\times \mathrm{Recall}}{\mathrm{Precision}+\mathrm{Recall}}$$

(4) $$AP=\int_{0}^{1}P(r)dr$$

(5) $$mAP=\frac{1}{N}\sum_{i=1}^{N}{AP}_{i}$$

where TP denotes a positive case that predicted true, FP denotes a positive case that predicted false, TN denotes a negative case predicted true, and FN denotes a negative case predicted false; AP denotes the area enclosed by the coordinates x and y of the precision–recall curve, and N denotes the number of detection categories; mAP@0.5 represents the mean value of AP for each class of detection target computed when the IoU threshold is set to 0.5; mAP@0.5:0.95 and the average value of the mAP were computed for each IoU threshold in steps of 0.05 from 0.5 to 0.95.

The multidimensional evaluation metrics and their technical significance are summarized in Table 2.

Moreover, the relative comparison metrics were calculated according to Equations (6)–(10).

(6) ∆mAP@0.5=mAP@0.5 of x, l or c model−mAP@0.5 of t or n model

(7) $$Params\;ratio=\frac{Params\;of\;l\;or\;c\;model}{Params\;of\;t\;or\;n\;model}$$

(8) $$FLOPs\;ratio=\frac{FLOPs\;of\;l\;or\;c\;model}{FLOPs\;of\;t\;or\;n\;model}$$

(9) $$Model\;size\;ratio=\frac{Model\;size\;of\;l\;or\;c\;model}{Model\;size\;of\;t\;or\;n\;model}$$

(10) $$FPS\;ratio=\frac{FPs\;of\;l\;or\;c\;model}{FPs\;of\;t\;or\;n\;model}$$

## 4. Results and Discussion

### 4.1. Model Comparison Results

The performances of different scales and versions of the YOLO model on the UAV-PDD2023 dataset were compared. The evaluation metrics are shown in Table 3.

**Figure 4:**
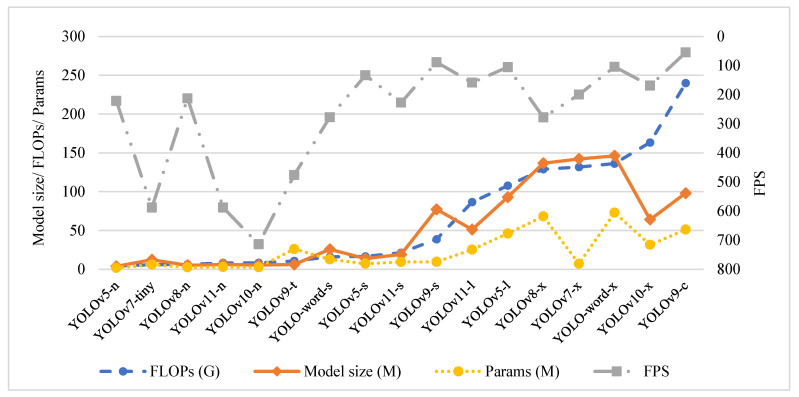
Comparison of efficiency evaluation results.

As can be seen from Table 2, YOLOv5-l and YOLOv9-c performed the best in detection accuracy, YOLOv5-l had a high mAP@0.5 of 89% and an F1 of 80.9%, while the second-placed YOLOv9-c had a mAP@0.5 of 82.2% and an F1 of 82%. Both models possessed a great number of parameters and high computational complexity. The inference efficiency of YOLOv9-c was the lowest among all models, followed by YOLOv9-s. In terms of model lightness, the YOLOv5-n, YOLOv8-n, and YOLOv10-n models demonstrated superior performance, with the YOLOv10-n achieving the highest detection efficiency of up to 714 FPS, followed by YOLOv7-tiny and YOLOv11-n. It is particularly noteworthy that YOLOv5s and YOLOv11-s presented some competitive advantages in terms of accuracy and lightweightness, indicating they may be suitable as baseline models for compression and application in embedded platforms. However, they performed poorly in efficiency, with FPS values of 133.3 and 227.3, respectively. A line graph was plotted in the order of computational sizes to further analyze the model’s ease of use and detection efficiency, as shown in Figure 4.

As demonstrated in Figure 4, there was a strong correlation between the number of model parameters and the model size, with the notable exceptions of the small and nano-models of YOLOv7 and YOLOv9, which are due to the incorporation of specific modules. Models demonstrating both lightness and high efficiency included YOLOv10-n, YOLOv7-t, YOLOv11-n, and YOLOv9-t, which may be suitable for scenarios with embedded real-time monitoring requirements.

**Figure 5:**
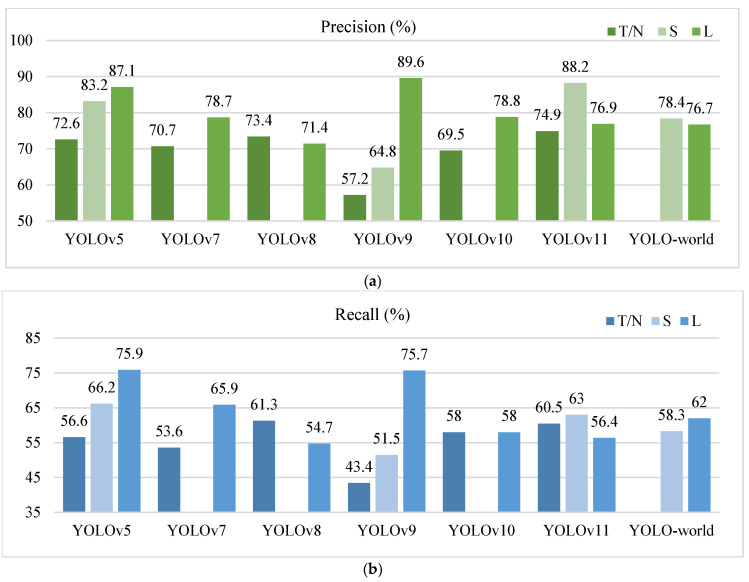
Comparison of precision and recall results. Note: nano and tiny (-n and -t) models are labelled as T/N; small-sized (-s) models are uniformly labelled as S, and large-sized (-c, -l, and -x) models are uniformly labelled as L.

To further visually compare the performance of the models in terms of precision and recall, comparison graphs were plotted as shown in Figure 5.

As shown in Figure 5, YOLOv9-c and YOLOv5-l showed excellent performance in terms of precision and recall, indicating that both models had good detection capability and detection accuracy, highlighting that these two model frameworks can be prioritized for application scenarios pursuing high-precision detection. However, their corresponding T/N versions of the model performed poorly. The high detection precision and detection rate of YOLOv11-s and YOLOv5-s, as well as a certain degree of model compression, made them potentially amenable to further improvement and application in embedded scenarios for UAV inspections. YOLOv8-n and YOLOv11-n also performed with relatively high detection precisions and recalls, coupled with their compacted model sizes, making them ideal for embedded applications that require high detection coverage. Among them, YOLOv11-n had the highest detection efficiency (FPS), which met the real-time requirements. Notably, YOLOv11-s exhibited a superb detection accuracy and detection rate compared to YOLOv11-l, which may be because the target in the dataset was either small or densely distributed. In such cases, the sense field design of the -s version may be more appropriate. In contrast, the -l version might be overly complex to optimize, potentially affecting performance negatively.

### 4.2. Balance Evaluation and Application Scenarios Analysis

**Figure 6:**
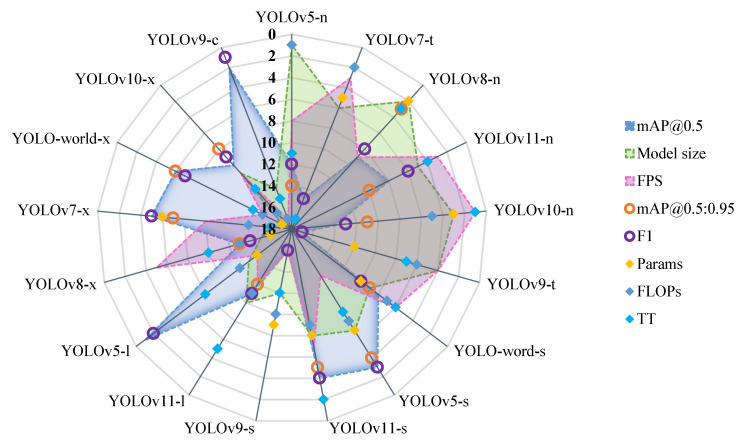
Comparison of efficiency evaluation results.

Based on the ranking of evaluation metrics (where 1 indicates optimal performance and 17 indicates the poorest performance), this study visualized model rankings through radar charts (Figure 6). Three core metrics were prioritized for comparative analysis: detection accuracy (mAP@0.5), detection efficiency (FPS), and model lightweight degree (model size).

As shown in Figure 6, the models exhibited significant performance variations:

(1) Single-Advantage Model Groups

YOLOv5-l, YOLOv9-c, YOLOv7-x, YOLOv5-s, and YOLOworld-x excelled in mAP@0.5 but lacked competitiveness in efficiency and lightweight metrics; YOLOv5-n and YOLOv8-n showed notable advantages in model size, while YOLv8-n demonstrated moderate detection accuracy.

(2) Dual-Advantage Balanced Group

YOLOv9-t, YOLOv11-n, and YOLOv7-t achieved a balance between model lightweightness and detection efficiency but exhibited relatively lower baseline accuracy.

(3) Comprehensive Excellence Group

YOLOv11-s delivered outstanding performances across all three metrics: detection accuracy (mAP@0.5 = 73.6%), efficiency (FPS = 227.3), and lightweightness (model size = 19.2 M), achieving the best overall performance. YOLO-wrorld1-s showed significant advantages in detection efficiency (FPS = 277.8) and model compression (model size = 25.8 M), with its mAP@0.5 at 66%.

This visualization revealed the trade-offs in the “accuracy–efficiency–lightweight trilemma”, providing multidimensional decision-making insights for model selection in engineering applications. Based on the established minimum detection accuracy threshold (mAP@0.5 > 60%), the efficiency-optimized benchmarks YOLOv10-n and YOLOv11-n warrant comprehensive validation for real-time vehicular/UAV detection systems. Conversely, the high-precision models YOLOv5-l and YOLOv9-c demonstrated particular suitability for security surveillance and industrial quality inspection scenarios. A further investigation into cross-environment generalization and multi-scale detection capability needs to be conducted. Notably, YOLOv11-s emerged as a balanced solution with competitive accuracy, efficient throughput, and compact architecture. This configuration presents strong potential for embedded deployment, particularly in edge computing devices and mobile systems.

### 4.3. Model Comparison Between Different Sized Models

A comparison was made between the performance metrics of the minimum (Max) model and the maximum model (Min) in Table 1. The comparison results are shown in Table 4.

As there were no changes in the main modules of different sizes in YOLOv5, YOLOv8, YOLOv11, or YOLO-world, the four groups were discussed first as Group 1, and the others were Group 2. The comparison of models of differing sizes in Table 3 reveals the following conclusions.

Group 1: (1) The size of the model and the FLOPs of the YOLOv5 changes were very significant; the l-model was 24–27 times the n-model, and the detection accuracy also underwent a significant enhancement with alterations to network depth and width. (2) YOLOv8-n exhibited no loss in accuracy, but rather a modest enhancement, despite the significant compression of the model (the size of the n-model was approximately 1/26 the size of the x-model) and a substantial reduction in the amount of computation (the FLOPs of the n-model were about 1/20 of the FLOPs of the x-model). However, YOLOv8-n achieved a decrease in FPS. (3) The YOLOv11-n model was compressed significantly, with the n version being compressed approximately 1/8 compared to the l version, and the FLOPs being reduced to about 1/11. Intriguingly, the s-version of the model attained the highest road distress detection accuracy (Table 2), which may be attributable to the overfitting problem engendered by the deeper and wider network of YOLOv11-l. (4) The two versions (x and s) of YOLO-world exhibited a minor disparity in detection accuracy, with the compression degree of the model being about 1/6 and the reduction in the FLOPs being 1/8.5.

Group 2: (1) The differences in YOLOv7 were mainly in the confusion and detector parts. The compression degree and the change in the accuracy of the YOLOv7 model were both significant. The mAP@0.5 of the t-model was improved by 13%, the size was 11.6 times that of the t-model, and the FLOP value was 22.6 times that of the t-model. (2) There was a slight difference between the backbone components of the t/s and c versions of YOLOv9; the detection accuracy of YOLOv9-t was significantly improved with an increase of 36.3% in mAP@0.5; the YOLOv9-t model’s FLOPs were 1/22.4 that of the c-model, and the model size was compressed to 1/16. Moreover, YOLOv9-t achieved the highest FPS increase. (3) Both components of the backbones and neck networks of YOLOv10-n and YOLOv10-x were different. Compared to YOLOv10-x, the YOLOv10-n model was compressed to approximately 1/12, and the FLOP value was reduced to approximately 1/20. However, the change in model accuracy in detecting highway distress using UAV data was not significant, with mAP@0.5 being reduced by 1.9%.

### 4.4. Comparison of Classification Detection Accuracy of Representative Models

**Figure 7:**
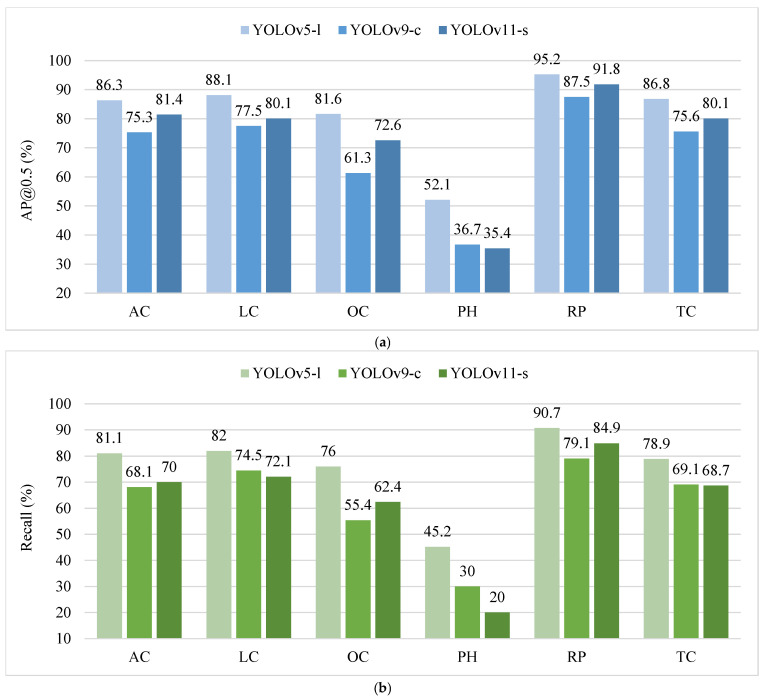

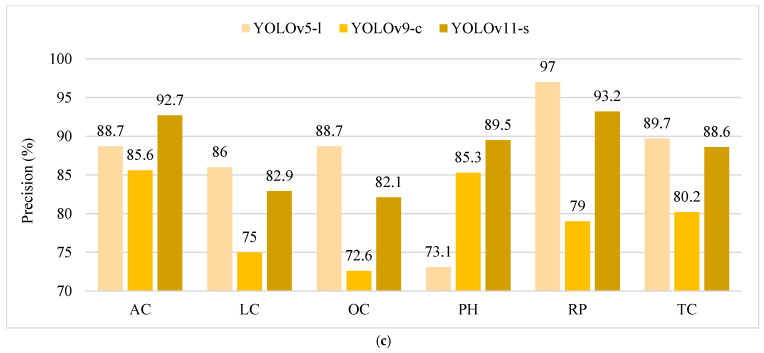
Comparison of classified detection accuracy. (**a**) AP@0.5; (**b**) Recall; (**c**) Precision.

The classified detection accuracy results of representative models including YOLOv5-l, YOLOv9-c, and YOLOv11-s were compared, as shown in Figure 7.

**Figure 8:**
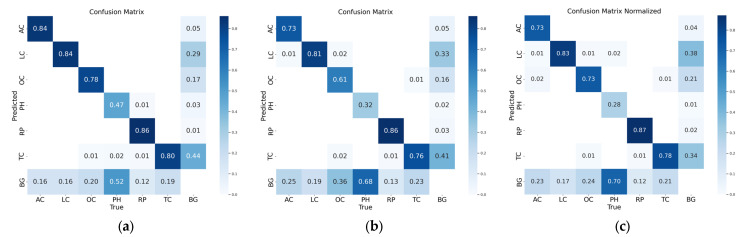
Confusion matrixes. (**a**) YOLOv5-l, (**b**) YOLOv9-c, (**c**) YOLOv11-s.

From Figure 7, it can be seen that different models performed differently in terms of classification detection accuracy, with potholes being the most difficult target to balance detection rate and detection precision. As shown in Figure 7a, YOLOv5-l achieved the highest detection accuracy for all highway distress types; YOLOv11-s outperformed YOLOv9-c in most categories, highlighting the efficacy of the smaller model. As shown in Figure 7b, YOLOv5-l exhibited an excellent detection ability in all distress types, higher than the other two models, with the lowest detection rate for pothole targets. The pothole detection rate of YOLOv11-s was extremely low with a recall of 20% and that of YOLOv9-c was as low as 30%, which reflects the difficulty in detecting small targets. From Figure 7c, it can be seen that the YOLOv5-l and YOLOv11-s models performed similarly in terms of precision, and none of them had an absolute advantage, but YOLOv9-c performed relatively poorly, probably because of a higher probability of false detection. To further analyze the problematic manifestations of detection anomalies in different disease categories, a confusion matrix was drawn, as shown in Figure 8.

As shown in Figure 8, potholes were the most likely to be missed, while the second category of easy-to-miss objects was diagonal cracks, while transverse and longitudinal cracks were mainly true-negative wrongly detected; YOLOV5-l had the best overall performance but presented the highest proportion of wrongly detected transverse cracks; no significant difference existed between YOLOv9-c and YOLOv11.

## 5. Conclusions

This paper conducted a comparative study on the comprehensive application of the YOLO series of algorithms in the UAV-based inspection of highway distress. The algorithms compared included YOLOv5-n, YOLOv5-s, YOLOv5-l, YOLOv7-tiny, YOLOv7-x, YOLOv8-n, YOLOv8-x, YOLOv9-t, YOLOv9-s. YOLOv9-c, YOLOv10-n, YOLOv10-x, YOLOv11-n, YOLOv11-s, YOLOv11-l, YOLO-word-s, and YOLO-word-x, and the main conclusions obtained are as follows.

(1) YOLOv5-l and YOLOv9-c achieved the highest detection accuracy (mAP@0.5, mAP@0.5:0.95, F1) on UAV highway inspection data. YOLOv5-l performed well in mean and classification detection precision and recall, while YOLOv9-c performed poorly in classification precision and recall.
(2) YOLOv10-n, YOLOv7-t, and YOLOv11-n achieved the highest detection efficiency; YOLOv5-n, YOLOv8-n, and YOLOv10-n had the smallest model sizes; and YOLOv11n was the model with the best performance in terms of combined detection efficiency (FPS), model size, and computational complexity (FLOPs), which is expected to be used for embedded real-time detection.
(3) It is evident that both the YOLOv5-s and the YOLOv11-s are capable of achieving a balance between the detection accuracy and the lightweight degree of the model; however, the efficiency is merely average at best. It can be concluded that the models may be considered suitable for lightweight detection platforms that have higher accuracy requirements.
(4) Comparing the t/n and l/c versions, it was found that the change of the backbone network in YOLOv9 had the greatest impact on the model detection accuracy, followed by the impact of the network depth_mulltiple and width_multiple of YOLOv5; the relative compression degrees of the models of YOLOv5-n and YOLOv8-n were the highest; and YOLOv9-t achieved the greatest efficiency improvement in UAV highway detection, followed by v10-n and v11-n.

The limitations of this study are as follows: (1) Tests were conducted on the same hardware platform, which precluded a comparative analysis of applications deployed on different platforms. This limits the generalizability of the findings across diverse hardware environments. (2) The study lacked validation using a diverse and extensive dataset. Additionally, there was insufficient research on the impact of data quality on the results. This may affect the robustness and applicability of the findings in broader contexts.

Suggestions for further research: (1) Investigate the stability of embedded terminal applications, particularly those utilizing preferred lightweight algorithms. This could provide insights into their reliability and performance in real-world scenarios. (2) Conduct studies aimed at improving the accuracy and generalization capabilities of high-precision detection applications. This would help enhance their applicability across various contexts and data types. (3) Test the performance of high-efficiency models in real-time monitoring scenarios. This could offer valuable insights into their feasibility and effectiveness for time-sensitive applications.

## Figures and Tables

**Table 1:** The framework composition for the comparison tests.

Model	Backbone	Neck	Head	Depth Multiple and Width Multiple
YOLOv5-n	CSPDarknet53, C3, SPPF	PANet	Conv, Upsample, Concat, Detect	0.33, 0.25
YOLOv5-s				0.33, 0.50
YOLOv5-l				1.0, 1.0
YOLOv7-tiny	Conv, Concat, Max Pooling	SPP	IDetect	0.33, 0.5
YOLOv7-x		SPPCSPC, Upsample, Concat		1.25, 1.25
YOLOv8-n	Conv, C2f, SPPF	Upsample, Concat, C2f	Detect	0.33, 0.25
YOLOv8-x				1.0, 1.25
YOLOv9-t	Conv, ELAN1, AConv, RepNCSPELAN4	SPPELAN, Upsample, Concat operation	DualDDetect	0.33, 0.25
YOLOv9-s				1.0, 1.0
YOLOv9-c	Conv, Silence, ADown, RepNCSPELAN4			1.33, 1.25
YOLOv10-n	Conv, C2f, SCDown, SPPF, PSA	Upsample, Concat, C2f	v10Detect	0.33, 0.25
YOLOv10-x	Conv, C2f, SCDown, C2fCIB, SPPF, PSA	Upsample, Concat, C2fCIB-module		1.33, 1.25
YOLOv11-n	Conv, C3k2, SPPF, C2PSA	Upsample, Concat, C3k2module	Detect	0.5, 0.25
YOLOv11-s				0.5, 0.5
YOLOv11-l				1.0, 1.0
YOLO-world-s	Conv, C2f, SPPF	Upsample, Concat, C2fAttn, ImagePoolingAttn	WorldDetect	0.33, 0.5
YOLO-world-x				1.0, 1.25

**Table 2:** Multidimensional evaluation metrics.

Type of Guideline	Specific Indicator	Engineering Significance
Accuracy	Precision	The reliability of the model’s prediction results and high precision means that the model has a low rate of FP problems.
	Recall	Reflecting the model’s ability to detect targets, a high recall rate means that it can find as many relevant targets as possible to avoid missing important information.
	mAP@0.5	Comprehensive detection capabilities
	mAP@0.5:0.95	A more comprehensive evaluation of the model’s efficacy under diverse precision requirements and necessitates enhanced robustness and accuracy of the model.
	F1	Suitable for scenarios that require both detection accuracy and completeness. A higher F1 score indicates that the model has achieved a better balance between precision and recall.
Efficiency	FPS	Real-time guarantee
	TT	An important measure of model training efficiency. A shorter TT means that the model can converge faster, thus reducing the cost of development and iteration.
Lightweight	Params	Reflects the complexity of the model; the higher the number of parameters, the more expressive the model is, but it may also lead to higher computational costs and storage requirements.
	FLOPs	Measure of a model’s computational complexity; lower FLOPs means the model is more computationally efficient and can achieve faster inference with limited computational resources.
	Model size	Directly affects the deployment cost and storage requirements; smaller models are better suited to run on devices with limited memory, such as mobile devices and embedded systems. In addition, model size affects model loading time and transfer efficiency.

**Table 3:** The evaluation results for the YOLO family.

Model	mAP@0.5 (%)	mAP@0.5:0.95 (%)	F1 (%)	Params (M)	FLOPs (G)	Model Size (M)	FPS	TT (Hours)
YOLOv5-n	63	31.6	64	**1.8**	**4.2**	**3.8**	221.4	2.3
YOLOv5-s	**76.2**	44.3	**76**	7.235	16.6	13.7	133.3	2.15
YOLOv5-l	**89.0**	**55.7**	**80.9**	46.135	107.7	92.9	105.3	1.736
YOLOv7-t	59.5	28.3	61	6.021	**5.82**	12.3	**588**	9.285
YOLOv7-x	72.5	41.4	71.8	7.081	131.7	142.2	200	5.880
YOLOv8-n	64.7	**45.1**	67	**2.583**	**6.3**	**5.2**	212.8	**0.72**
YOLOv8-x	61.7	32.8	61.9	68.129	128.99	136.7	278	2.178
YOLOv9-t	45.9	21.5	52.2	26.19	10.7	6.1	476	1.7
YOLOv9-s	53.6	26.8	56	9.601	38.7	77.2	88.5	2.38
YOLOv9-c	**82.2**	**57.9**	**82**	51.182	239.9	98.1	54.85	3.17
YOLOv10-n	61.6	35.7	63	**2.686**	8.2	**5.5**	**714.3**	**0.55**
YOLOv10-x	63.5	39.6	66.8	31.585	163.4	64.1	169	2.511
YOLOv11-n	66	35.9	69.6	3.01	8.1	6.3	**588**	0.92
YOLOv11-s	73.6	42.7	73.3	9.415	21.3	19.2	227.3	**0.677**
YOLOv11-l	63.1	34.4	65	25.283	86.6	51.2	158.7	1.196
YOLO-word-s	66	36	66.6	12.749	16.02	25.8	277.8	1.491
YOLO-word-x	67.3	42.5	68.6	72.856	136.23	146.2	104.2	2.525

Note: bolded are the top three performance metric results.

**Table 4:** The comparison results between the Max- and Min-sized models.

Model	∆mAP@0.5 (%)	Params Ratio (M)	FLOPs Ratio (G)	Model Size Ratio (M)	FPS Ratio
YOLOv5	26	25.6	25.6	24.4	1/2
YOLOv7	13	1.2	22.6	11.6	1/3
YOLOv8	−3	26.4	20.5	26.3	11/3
YOLOv9	36.3	2.0	22.4	16.1	1/9
YOLOv10	1.9	11.8	19.9	11.7	1/4
YOLOv11	−2.9	8.4	10.7	8.1	1/4
YOLOv-world	1.3	5.7	8.5	5.7	3/8

## Data Availability

The raw data supporting the conclusions of this article have been cited in the article. https://zenodo.org/records/8429208 (accessed on 20 February 2025).
